# 3D Finite Element Electrical Model of Larval Zebrafish ECG Signals

**DOI:** 10.1371/journal.pone.0165655

**Published:** 2016-11-08

**Authors:** James Crowcombe, Sundeep Singh Dhillon, Rhiannon Mary Hurst, Stuart Egginton, Ferenc Müller, Attila Sík, Edward Tarte

**Affiliations:** 1 School of Engineering, University of Birmingham, Birmingham, United Kingdom; 2 Institute of Cancer and Genomic Sciences, College of Medical and Dental Sciences, University of Birmingham, Birmingham, United Kingdom; 3 Institute of Clinical Sciences, College of Medical and Dental Sciences, University of Birmingham, Birmingham, United Kingdom; 4 School of Biomedical Sciences, Faculty of Biological Sciences, University of Leeds, Leeds, United Kingdom; National Institutes of Health, UNITED STATES

## Abstract

Assessment of heart function in zebrafish larvae using electrocardiography (ECG) is a potentially useful tool in developing cardiac treatments and the assessment of drug therapies. In order to better understand how a measured ECG waveform is related to the structure of the heart, its position within the larva and the position of the electrodes, a 3D model of a 3 days post fertilisation (dpf) larval zebrafish was developed to simulate cardiac electrical activity and investigate the voltage distribution throughout the body. The geometry consisted of two main components; the zebrafish body was modelled as a homogeneous volume, while the heart was split into five distinct regions (sinoatrial region, atrial wall, atrioventricular band, ventricular wall and heart chambers). Similarly, the electrical model consisted of two parts with the body described by Laplace’s equation and the heart using a bidomain ionic model based upon the Fitzhugh-Nagumo equations. Each region of the heart was differentiated by action potential (AP) parameters and activation wave conduction velocities, which were fitted and scaled based on previously published experimental results. ECG measurements *in vivo* at different electrode recording positions were then compared to the model results. The model was able to simulate action potentials, wave propagation and all the major features (P wave, R wave, T wave) of the ECG, as well as polarity of the peaks observed at each position. This model was based upon our current understanding of the structure of the normal zebrafish larval heart. Further development would enable us to incorporate features associated with the diseased heart and hence assist in the interpretation of larval zebrafish ECGs in these conditions.

## Introduction

Sudden cardiac death accounts for more than more than 450,000 deaths every year in the USA alone [1]. Amongst the most common heart problems are cardiac arrhythmias and QT prolongation (increased delay between ventricular depolarisation and repolarisation). In order to increase the understanding of these dysfunctions it is necessary to study the electrical properties of the heart during early development. Over the last ten years the zebrafish has been increasingly used to study these dysfunctions and has been suggested as a useful model for studies of human cardiac electrophysiology and development [2]. This is due to the surprisingly close electrophysiological similarity between human and zebrafish hearts. The shape and duration of zebrafish myocyte action potentials (APs) are largely similar to those of human myocytes as orthologues of the cardiac ion channels found in humans also exist in zebrafish e.g. zERG (orthologue of human hERG) [3]. In addition, ECG morphologies are also similar and so are QT intervals and heart rates (120–180 beats per minute (bpm)) [4–6]. The zebrafish has a simple circulatory system with a two chambered heart. At 3 days post fertilisation (dpf) the heart is approximately 100 μm in size and contains ~300 cells [7], each with an area of ~300 μm^2^ [8] that are coupled by gap junctions [8, 9]. Cardiac activity can be assessed quickly following gene mutation in comparison to other animal models, as zebrafish development occurs very rapidly with a beating heart present at 24 hours post fertilisation (hpf) [10, 11]. As their ECG is similar to mammals and they respond very similarly to drug treatment as humans, zebrafish embryos and larvae can be useful screening tools with several additional benefits. These include the small amount of chemicals necessary for drug testing, fast and inexpensive embryo production, rapid generation of transgenic animals and fewer regulatory restrictions in care and maintenance [1, 6].

The ECG is a time-resolved vector measure of electrical activity of the heart at the body surface and is one of the most important diagnostic tools in assessing cardiac dysfunction and evaluating potential drug side effects. Both adult and larval zebrafish ECGs have been measured at various stages of development [1, 6, 12–16] and changes in the QT interval have been observed following targeted drug treatment [6].

Computational models provide a platform for investigating aspects of physiology that are difficult to study within intact organisms for both ethical and/or practical reasons, such as animal welfare and costs. Despite being computationally intensive these models have become increasingly realistic and informative. They have begun to be more widely used due to the greater availability and increase in computing power with models of the heart even being constructed for individual patients to aid diagnosis [17]. Simulations of the heart can address its electrical or mechanical properties and current models incorporate either or both of these aspects [18–20].

Models of the heart range in scale from a single cell [21], sheets of tissue [22], a partial heart [23, 24], to a whole heart [25–27]. This paper focuses on simulating the electrical activity of the zebrafish heart and thus determining the waveform of the ECG which is known as the forward problem of electrocardiography [28–30]. Whilst the mechanical properties of the heart may affect the resultant ECG (see [20]), the relationship between electrical and mechanical properties of the heart increases model complexity and hence computational intensity.

As most cardiac research is conducted with the aim of developing therapies for human diseases, the majority of previous work in this field focuses on the human heart. Models that incorporate the human body have been generated to investigate ECG generation as well as its parameters, including drug effects [31, 32], although some modelling using other mammalian hearts (e.g. sheep, dog, etc.) is also available [33, 34]. Some models have even been made of fish hearts, e.g. a fluid flow model of the larval zebrafish heart [35] and a mechanical model of the carp heart [36], but no electrical simulations exist for the zebrafish larva.

This paper introduces an electrical model of the zebrafish larval heart and body, with the aim of simulating the ECG and making predictions to aid in functional measurements. At present it can simulate the activation wave and produce an ECG that is comparable to those measured *in situ*. The predictions it can provide are where the largest magnitude signal should be recorded with electrodes, and what shape of ECG waveform should be expected at different positions. The structure of the model is explained followed by a comparison to *in vivo* ECG measurements obtained from 3 dpf zebrafish larvae.

## Materials and Methods

### Model geometry

**Fig 1:**
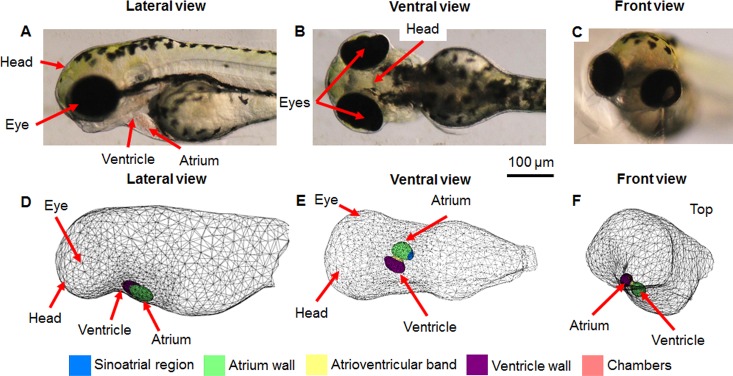
Comparison of 3D model geometry to 3 dpf zebrafish anatomy. A) lateral, B) ventral, C) front brightfield views; D) lateral, E) ventral F) front view of model. Different coloured regions highlight the distinct heart regions within the model.

**Fig 2:**
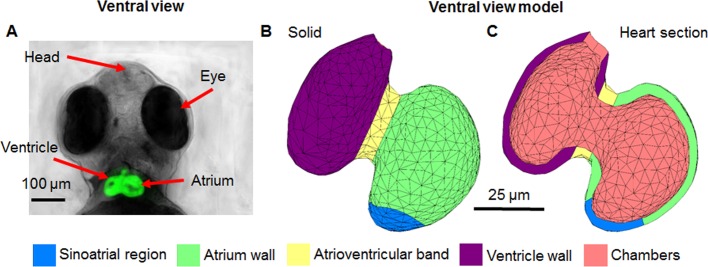
Image of 3 dpf zebrafish heart compared to model heart geometry. All images are from a ventral view. A) depiction of a 3 dpf *Tg(fli-1*:*EGFP)* zebrafish with a fluorescing heart, B) solid view of heart geometry, C) model heart geometry cut through showing the distinct regions.

A 3D geometry model was constructed from a combination of sources including an image stack generated using Optical Projection Tomography (OPT) [37, 38], images of a 3 dpf zebrafish body [39] and heart [7, 40–51], as well as our own bright field microscopy images. Slicer [52, 53] was used to visualise the image stack and extract the main shape of the zebrafish which was then imported into Blender (Blender Foundation [4]) where it was edited to match reference images, and the final shape was then imported into COMSOL Multiphysics (COMSOL Inc.) for electrical simulation. The geometry of the model consisted of two main parts, the zebrafish body and the zebrafish heart (**Fig 1** and **Fig 2**).

### Electrical model

The electrical activity was simulated using a combination of Laplace’s equation and the bidomain equations. The body was treated as a homogenous volume conductor described by the Laplace equation:

$$\nabla ∙(-\sigma_{b}\nabla V)=0$$ (1)

Where V is the voltage in the body and σ_b_ is body conductivity.

The cardiac electrical activity was simulated using the bidomain equations that have been widely used to model the human heart [27, 29, 32]. The heart is treated as a region consisting of two interpenetrating domains representing the intracellular and extracellular spaces. A derivation of the equations is given in **S1 File**. The following equations were inputted into COMSOL:

$$\frac{\partial V_{e}}{\partial t}-\frac{\partial V_{i}}{\partial t}=\frac{1}{C_{m}A_{m}}(-\nabla ∙(-\sigma_{e}\nabla V_{e})+A_{m}i_{ion})$$ (2)

$$\frac{\partial V_{i}}{\partial t}-\frac{\partial V_{e}}{\partial t}=\frac{1}{C_{m}A_{m}}(\nabla ∙(-\sigma_{i}\nabla V_{i})-A_{m}i_{ion})$$ (3)

Where V_e_ is the extracellular voltage (V), V_i_ is the intracellular voltage (V), σ_e_ is the extracellular conductivity (S/m), σ_i_ is the intracellular conductivity (S/m), i_ion_ is the ionic current (A/m^2^), A_m_ is the surface-to-volume ratio (1/m) and C_m_ is the membrane capacitance per unit area (F/m).

There are many different ionic current models of cardiac cells, ranging from realistic with equations for each ion channel [21, 26, 54] to phenomenological models where just the main properties are described, such as the Mitchell and Schaeffer model [55] and the Fitzhugh-Nagumo (FHN) equations [56]. Both of these models have been previously used to simulate the ECG [32, 57, 58].

The Rogers-McCulloch version of the FHN equations [59] used in this work are a modified version of the originals [56], and produce an AP more like those found in cardiac cells. Despite being phenomenological in origin, these equations were chosen as they reproduce the main properties of cardiac tissue (APs, wave propagation and threshold) and simulate the ECG [58] whilst being computationally inexpensive and not requiring measurements of individual ion channels from the zebrafish heart. i_ion_ in its self-excitatory form is given by:

$$i_{ion}=kc_{1}(V_{m}-B)\left[a-\frac{\left(V_{m}-B\right)}{A}\right]\left[1-\frac{\left(V_{m}-B\right)}{A}\right]+kc_{2}u$$ (4)

in the sinoatrial region and by:

$$i_{ion}=kc_{1}(V_{m}-B)\left[a-\frac{\left(V_{m}-B\right)}{A}\right]\left[1-\frac{\left(V_{m}-B\right)}{A}\right]+kc_{2}u(V_{m}-B)$$ (5)

in the rest of the heart. Vm is the transmembrane potential given by:

$$V_{m}=V_{i}-V_{e}$$ (6)

Where V_i_ is the intracellular voltage and V_e_ is the extracellular voltage. *u* is a recovery variable given by:

$$\frac{\partial u}{\partial t}=ke\left[\frac{\left(V_{m}-B\right)}{A}-du-b\right]$$ (7)

k, c1, c2, A, B, a b, d and e, are parameters that effect the shape of the AP. Each parameter has a major effect on the AP shape with interactions limited between parameters. The original FHN equations used arbitrary space, time and voltage units. The scaling factors k (time) and A (voltage) which were introduced scale the APs produced so that 1 time unit = 1 second and 1 voltage unit = 1 volt [58]. B offsets the voltage to set the resting potential, while c1 and c2 affect the upstroke/down stroke gradient. ‘a’ and ‘e’ affect the action potential duration (APD).

### Boundary conditions

A reasonable assumption is that the exterior faces of the body are insulated, and there is a ground point at the tail end. The boundaries between the body and heart then have the condition:

$$V=V_{e}$$ (8)

All boundaries between the heart (excitable tissue) and the body/chambers (volume conductor) have a zero flux condition for V_i_:

$$-n∙\sigma_{i}\nabla V_{i}=0$$ (9)

where n is the unit vector normal from the boundary. For V_e,_ the inward flux is equal to the outward current density **J** from the body:

$$-n∙\sigma_{e}\nabla V_{e}=n∙J$$ (10)

### Use of COMSOL software

The finite element method (FEM) was used to solve the equations in the COMSOL Multiphysics environment. The models were solved from 0 to 0.5 seconds to simulate one cardiac cycle with results displayed at 1 ms intervals using the back differentiation formula (BDF) algorithm within COMSOL. The model consisted of 85,255 tetrahedral elements with 152,630 degrees of freedom. Each simulation took approximately 20 minutes to complete on a PC with an i7 processer and 16 GB of RAM.

### Zebrafish maintenance and embryo collection

For all ECG recording experiments wild-type Tübingen strain zebrafish were used with the *Tg(fli-1*:*EGFP)* line used for imaging. Zebrafish lines were maintained under standard conditions in compliance with the UK Animals (Scientific Procedures) Act of 1986 in a flow-through system of aerated, charcoal filtered tapwater with a 12 hour light/dark cycle (Tecniplast; UK). To obtain adequate samples, breeding pairs were set up in breeding cages the day before embryo collection. The following morning zebrafish embryos obtained from the crosses were transferred to E3 embryo medium (Sigma-Aldrich), which was changed on a daily basis and maintained at a temperature of 28°C. Zebrafish 3 dpf of age were used for all ECG experiments so no ethical approval was required.

### *In vivo* ECG recording

For all experiments, 3 dpf zebrafish larvae were used. They were first anaesthetised in ethyl-3-aminobenzoate methanesulfonate (0.3 mg/ml; Sigma-Aldrich) for 5–10 minutes before being transferred into 3 mL of E3 embryo medium in the plate used for subsequent measurements. Once anaesthetised, individual zebrafish larvae was then transferred to the ECG recording plate (mini Petri dish containing a 2% agarose layer with grooves). The larva was positioned ventrally within an agarose groove and the tip (2 μm diameter) of a borosilicate glass micropipette (P84, World Precision Instruments) was positioned on the skin surface above the ventricle, the atrium, or above the atrioventricular band (avoiding penetration) using micromanipulators (Narishige) and Inchworm step motors (Burleigh), viewed under a Nikon microscope (SMZ600). The micropipettes were filled with 3M potassium acetate solution (Sigma-Aldrich) using MicroFil (P85, World Precision Instruments, USA), and a chloridised silver wire was inserted to carry electrical signal to the amplifier. A second reference electrode was placed in the surrounding medium during recordings. The differential amplifier (NPI electronics, Germany) used for recording was operated in DC mode with the high pass filter set at 0.1 Hz. The raw ECG signals were digitised (PowerLab; ADI Instruments) and viewed using LabChart 7 (ADI Instruments).

All of the recording equipment was housed on an air table within a grounded Faraday cage to minimise background noise. Experiments were performed at 28°C and the temperature was controlled using a sensor placed in the recording plate and a homemade heating element (Cryocon 24 temperature controller, USA).

## Results

### 3D Geometry model

The model was based on a 3 dpf zebrafish as this is the stage generally used for ECG recordings because the heart is functioning normally and positioning of the zebrafish is easier due to the lack of a swim bladder. It may be observed that the model is an accurate representation of the larval zebrafish (**Fig 1A, 1B and 1C**) when compared to the model geometry (**Fig 1D, 1E and 1F**). The heart is located very close to the surface and it is visible through the skin from a lateral position, and also from above in a ventral position, as replicated within the model. The model heart was approximately 100 μm in size with ~7 μm walls.

The heart geometry was split into five distinct regions based on different sections of the zebrafish heart: the sinoatrial region, atrium wall, atrioventricular band, ventricle wall and heart chambers (**Fig 2**).

### Model parameters

As is the case in the human heart, each region within the zebrafish heart has distinct AP waveforms and conduction velocities that are essential to the integrated organ function [9]. In order to replicate these differences within the model the parameters used to generate APs and wave propagation were investigated to determine which values generated an AP most closely resembling those observed values.

**Fig 3:**
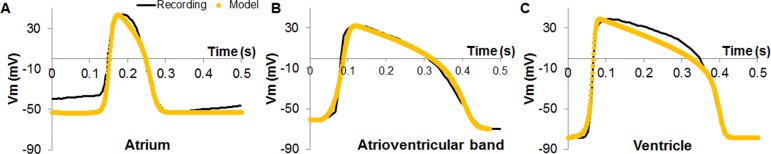
Comparison between recorded action potentials [9] from a 2 dpf heart and model action potentials. A) atrium B) atrioventricular band C) ventricle. Action potentials were recorded from explanted 2 dpf hearts at room temperature using patch pipettes and current clamp techniques.

Starting with the parameter values given in [58] a, b, c1, c2, e, k, A and B were changed systematically. A range of each parameter was used to determine its effect on the shape of the AP then the value that was found to give the closest match to the *in situ* recordings [9] was chosen as the final value. The closest match was determined by comparisons between the model output and the experimental recordings for the key parameters APD, magnitude, upstroke gradient and downstroke gradient. The major effect of each parameter on the shape of the AP was adjusted for one by one then the minor deviations on other AP parameters were accounted for by compensating in the preceding parameters. The APD was fitted by changing ‘e’ rather than ‘a’ as in [52]. For example, **S1 Fig** shows the effect changing parameter ‘e’ has on action potential duration (APD): decreasing ‘e’ increases APD and *vice versa*. The final parameter values are shown in **Table 1** and initial input values are shown in **Table 2**. The APs produced by the parameters are compared to the recorded APs in **Fig 3**. An AP for the sinoatrial region was not given in [9] so the parameters used in this region were chosen based on a combination that triggered the activation wave in the atrium with the alternative self-excitatory equation for i_ion_ (Eq 4).

**Table 1:** Final parameter values used within the model.

Parameter	SAR	Atrium	AV Band	Ventricle
**a**	-0.5	0.13	0.13	0.13
**b**	0.4	0	0	0
**c1**	0.182	0.572	0.234	0.572
**c2**	1	0.5	0.7	0.5
**d**	1	1	1	1
**e**	0.0001	0.004	0.00032	0.0011
**A**	0.2	0.1088	0.1085	0.1205
**B**	-0.135	-0.053	-0.07	-0.075
**k**	1000	1000	1000	1000
**Am (m**^**-1**^**)**	90000	90000	90000	90000
**Cm (Fm**^**-2**^**)**	0.01	0.01	0.01	0.01

Key: SAR = sinoatrial region; Atrium = atrial wall muscle; AV band = atrioventricular band; Ventricle = ventricle wall muscle

**Table 2:** Initial conditions.

Parameter	SAR	Atrium	AV Band	Ventricle
**Vi** (V)	-0.0500	-0.0530	-0.0585	-0.0750
**Ve** (V)	0	0	0	0
**u**	0	0	0	0

Key: SAR = sinoatrial region; Atrium = atrial wall muscle; AV band = atrioventricular band; Ventricle = ventricle wall muscle

A_m_ was calculated from results given in [8] for the average myocyte surface area (300 μm^2^) and the total myocardial volume (1x10^6^ μm^3^) in a 54 hpf zebrafish as well as the approximate number of cells in a 3 dpf zebrafish heart (300) [7]. The value of C_m_ was chosen in line with previous studies [60]. These values are shown in **Table 1** and all model parameters are combined in **S1 Table**.

The properties of both the model and recorded APs were then compared (see **Table 3**). The parameters of the model were selected so that the APD and magnitude of all three model APs best replicated the values of the *in situ* recordings (one for each region from [54]). The maximum gradient of the AP upstroke was lower than the recorded values, as was the maximum gradient of the downstroke, except for the atrium. The significance of each comparison is discussed later.

**Table 3:** Comparison between model and recorded action potential properties.

Region	Source	APD (ms)	Magnitude (mV)	Max upstroke (mV/ms)	Max down stroke (mV/ms)
**Atrium**	Recording	102	80.1	8.38	-3.99
	Model	108	96.8	4.59	-1.81
**AV band**	Recording	301	102.3	4.81	-0.65
	Model	316	103.4	2.28	-0.71
**Ventricle**	Recording	324	117.6	7.15	-1.69
	Model	329	116.4	4.16	-1.70

The value of each variable is given for both model and recorded action potential.

Key: SAR = sinoatrial region; Atrium = atrial wall muscle; AV band = atrioventricular band; Ventricle = ventricle wall muscle

### Model conduction velocity

In the model, one of the key parameters (along with AP upstroke/surface-to-volume ratio and capacitance) which determines the conduction velocity at which the APs travel through the heart is the conductivity. A scaling factor needed to be introduced to reduce the conductivity (increase resistivity), and hence the conduction velocity of each heart region (in comparison to [58]) in order to match measured velocities (from [61]), in the range of a 10^−2^ decrease from the values used in [58]. This is similar to the difference in the conduction velocities between the adult human heart and the larval zebrafish heart (see **Table 4**).

**Table 4:** Activation wave conduction velocities in an adult human and a 3 dpf zebrafish heart.

	Human	Zebrafish larvae (3dpf)	Model
**Atrium (mm/s)**	800->1000	1.3–2*	1.5
**AV Band (mm/s)**	20->50	0.1–0.3*	0.1
**Ventricle (mm/s)**	300->800	4->12	6.6

Measurements obtained from [30] (human) and [61] (zebrafish larvae). The range of values are associated with anisotropy in the tissue.

*approximation taken from activation map image.

The value of model conductivity needed to scale conduction velocity to measured results was found by comparison with the published activation map [61] for a 3 dpf zebrafish larval heart by comparing activation times for each region, which was taken to be the time interval between the wave front entering the region and leaving the region. This was ~32.5 ± 5 ms for the atrium, ~27.5 ± 5 ms for the AV band and ~10 ± 5 ms for the ventricle. An isotropic conductivity was used and scaled so that the activation times for each region within the model agreed with the activation map/measured velocities. This method was used along with estimated velocity equalisation (from the activation map) due to the lack of direct conduction velocity measurements in the atrium and AV band regions of the zebrafish heart. This resulted in the conduction velocities shown in **Table 4** which are within the measured ranges. The final conductivity values are shown in **Table 5** for each heart region as well as for the body and heart chambers.

**Table 5:** Conductivity values used within the model for each heart region.

Parameter	SAR	Atrium	AV Band	Ventricle	Body	Chambers
**σe** (Sm^-1^)	1.81E-05	1.81E-05	2.43E-06	1.55E-05	-	-
**σi** (Sm^-1^)	1.81E-05	1.81E-05	2.43E-06	1.55E-05	-	-
**σb** (Sm^-1^)	-	-	-	-	0.2	-
**σ**c (Sm^-1^)	-	-	-	-	-	0.7

Key: SAR = sinoatrial region; Atrium = atrial wall muscle; AV band = atrioventricular band; Ventricle = ventricle wall muscle; σ_b_ = body conductivity; σ_c_ = chamber conductivity

**Fig 4:**
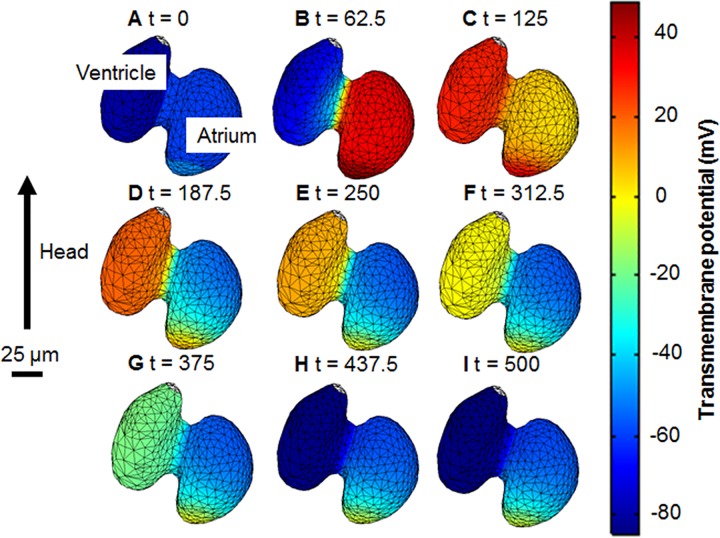
Temporal sequence of transmembrane potential (V_m_). The propagation of an action potential through the heart is shown at different times. Nine time steps were chosen to show the progression of the action potentials through all stages of the cardiac cycle. A) the heart at rest B) atrial depolarisation C) ventricular depolarisation D) the end of atrial repolarisation and ventricular plateau stage E,F,G,H) ventricular repolarisation I) returning to the rest state. The wave originates at the sinoatrial region then it progresses across the atrium, through the atrioventricular band and into the ventricle. An animation is given in **S2 File**.

Propagation of the AP through the model heart (**Fig 4**) begins in the sinoatrial region then moves into the atrium where it spreads; from here it travels through the atrioventricular band into the ventricle. **Fig 4** shows an overview of the activation, an animation of Vm over time is given in S2 File, which shows the full wave propagation.

### Model comparison to ECG measurements

**Fig 5:**
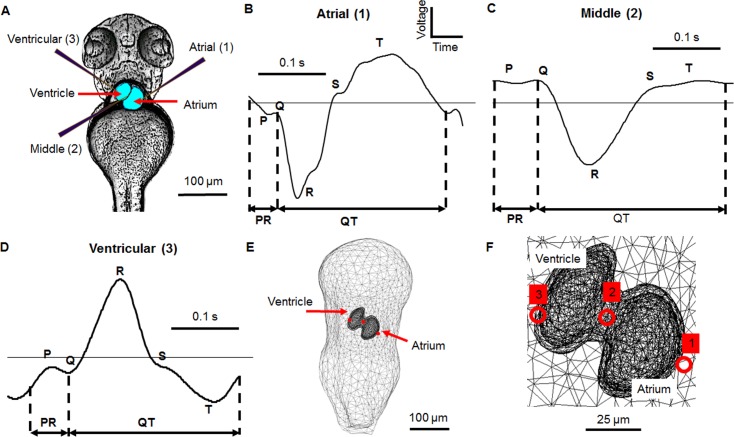
Electrode positions used for electrocardiogram recording with the corresponding measurements and model positions in a 3 dpf zebrafish. A) Processed image in ventral view showing electrodes in different recording positions. Electrode 1 is positioned over the atrium, electrode 2 over the atrioventricular band and electrode 3 over the ventricle, B) A representative atrial recording showing the key ECG features: P wave, QRS complex, T wave, PR interval and QT interval, C) A representative middle recording, D) A representative ventricular recording, E) Expanded ventral view of equivalent electrode positions within the model, F) Close-up ventral view of electrode positions over the heart. The signals from positions 1 and 3 were measured at the same time on the same zebrafish, that from position 2 was measured on a different animal and hence no voltage scale is shown. A positive deflection in the ECG is caused by the depolarisation wave moving towards the recording electrode

The ECG generally consists of three main components: the P wave (atrial depolarisation), the R wave (ventricular depolarisation) and the T wave (ventricular repolarisation) (**Fig 5**). The same gross features are observed in human, adult zebrafish and larval zebrafish ECGs [1, 6, 30], which enable zebrafish ECGs to be analysed using similar parameters to human ECGs. Common ECG parameters that are affected by cardiac diseases or drug treatments are the PR interval (which reflects the delay caused by the atrioventricular band), the QT interval (which is the interval between ventricular depolarisation and repolarisation) and the QRS complex (which is the depolarisation duration of the ventricle).

As with human electrocardiograms, the morphology of the zebrafish ECG signal was dependent on position of the recording electrode (**Fig 5B, 5C and 5D**) as demonstrated by three electrode positions: over the atrium (1), atrioventricular band (2) and ventricle (3) (**Fig 5A**). In order to investigate whether the model could replicate the measured changes in morphology, three points on the surface of the model body were chosen that corresponded to the electrode recording positions (**Fig 5E and 5F**). The direction the activation wave travels determines the polarity of the peaks seen in the ECG [30]. During depolarisation movement of the wave towards the recording electrode produces a positive peak in the ECG and when the wave moves away it produces a negative peak. For repolarisation the opposite is true with a negative peak produced when the wave travels towards the recording electrode and a positive peak is produced when the wave travels away. The magnitude of the peaks is also affected by the motion of the wave, with recording electrodes placed directly in the path of the wave measuring larger signals and *vice versa*.

**Fig 6:**
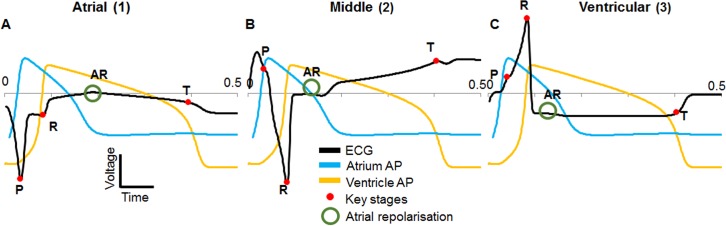
Model ECG at different positions with overlaid action potentials. A) atrial position, B) middle position, C) ventricular position. Red dots mark the key stages in each ECG (P, R and T waves). Atrial repolarisation is also marked (AR, green circle). No voltage scale is shown as the action potentials were scaled so that the ECG was visible on the same axes.

Firstly, the model ECGs were investigated without a comparison to recordings to confirm which stage of activation the peaks seen in the model ECG corresponded to (**Fig 6**). Both upstroke (depolarisation) and downstrokes (repolarisation) of APs were found to cause a peak in the ECG as expected. By overlaying the model APs the origin of a peak could be identified by noting which AP upstroke or downstroke it coincided with. For example, to confirm which peak was the P wave (atrial depolarisation) the model atrium AP was overlaid onto the model ECG. The peak that occurred at the same time as the atrium upstroke was then taken as the P wave. The same process was used for the R and T waves confirming that all the major components expected to be found in an ECG signal from a zebrafish larva were present in the model. Other peaks were also seen corresponding to atrial repolarisation (marked as AR).

As expected from unipolar records, the model ECG shape was found to vary across the surface of the zebrafish body, with a larger P wave over the atrium, and the R and T waves larger over the ventricle. The polarity of each ECG peak varied across the surface of the body over the heart; a positive peak refers to a maximum or positive deflection in the ECG and a negative peak refers to a minimum or negative deflection in the ECG whether the peak is situated above or below zero volts.

Movement of the depolarisation wave through the atrium, atrioventricular band and ventricle away from the atrial electrode position caused negative P and R waves; repolarisation had the opposite effect including a positive T wave. Similarly, the middle position showed a positive P wave, negative R wave and positive T wave. The ventricular position was positive P and R waves with a negative T wave (**Fig 6**).

### Comparison of model output with ECG recordings

**Fig 7:**
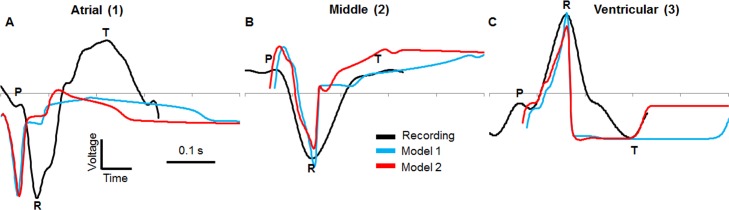
Comparison between ECG recordings and model ECG. The recorded ECG at three positions was compared to the model output. A) model comparison to atrial recording, B) model comparison to middle recording and C) model comparison to ventricular recording. Model 1 has fitted action potential durations, model 2 has a reduced action potential duration that gives a similar QT length to the recordings. No voltage scale is shown as the model ECG voltages are much smaller than the recordings.

The model ECGs from the atrial, middle and ventricular positions were then compared to *in vivo* recordings from similar positions (**Fig 7**). Initially, QT interval of the model ECGs (Model 1) was much longer than the QT interval of recordings, likely due to differences in temperature and the age of the fish as QT interval is mainly dependent on the duration of the ventricle AP. The APs were recorded at room temperature with 2 dpf zebrafish [9] and the ECGs were recorded at a higher temperature of 28°C with 3 dpf zebrafish. APDs are temperature dependent in the adult zebrafish [62], with higher temperatures reducing APD but with no major change in AP shape. Therefore, the difference in QT lengths between the model ECGs and recorded ECGs was reduced by decreasing the model APD of all APs using parameter ‘e’ (**S1 Fig**) so that the APD of the ventricle AP was approximately equal to the QT length of the recorded ECGs (Model 2), thus giving the model ECGs similar QT lengths to the recordings. The model 2 APs are shown in comparison to the model 1 APs in **S2 Fig**.

The voltage of ECG recordings were scaled to the model size for comparison. Relative magnitudes between R and T are more relevant than absolute magnitudes due to the amplification of the measured signal, multiple recorded signals and the reduced voltage seen in the model because of the conductivity scaling factor and the high surface-to-volume ratio (model ECGs peaks were in the 1x10^-10^ V range).

For the atrial position both model and recording show negative P and R waves with a positive T wave; atrial repolarisation is also seen in the model. In the middle position a positive P wave is seen for both model and recording, with a negative R wave and positive T wave for both. The ventricular position shows similar agreement with a positive P wave and negative R and T waves.

Two of the common ECG parameters that are used to assess drug effects are the PR interval (measured from the start of the P wave to the start of the Q wave) and QT interval (from the start of the Q wave to the end of the T wave). A comparison between model ECG parameters and measured ECG parameters from the three positions showed variable agreement (**Table 6**). Position 2 shows the best agreement between measurement and recording for the P width with widths of 25 ms for the recording and 41 ms for the model. The T wave width is best replicated at position 2 with a width of 34 ms for the recording and 33 ms for the model. The QT interval is best replicated at position 3 with 232 ms for the recording and 204 ms for the model. Position 3 also shows the best agreement with the R wave width.

**Table 6:** A comparison between model ECG (model 2 from Fig 8) parameters and measured ECG parameters.

Region	Source	P width (ms)	R width (ms)	T width (ms)	PR (ms)	QT (ms)
**Atrial (1)**	Recording	27	91	147	27	250
	Model 2	49	38	41	49	193
**Middle (2)**	Recording	25	157	34	69	244
	Model 2	41	55	33	41	209
**Ventricular (3)**	Recording	40	117	76	55	232
	Model 2	20	53	47	20	204

The value of each variable is given for both model and recorded ECG.

The relative peak amplitude at each position were also compared (**Table 7**), determined from the start to the peak for the P wave, and from the Q wave start to the peak of the R wave for the R wave. The relative peak heights for each recording position and the model was compared. P to R was found to be best replicated in position 3 (8.4 measured and 3.7 model), R to T in position 3 (4.42 measured and 2.47 model) and P to T also in position 3 (0.53 measured and 0.67 model).

**Table 7:** A comparison between model relative peak heights and measured relative peak heights.

Region	Source	P to R	R to T	P to T
**Atrial (1)**	Recording	10.83	2.29	0.21
	Model 2	0.01	0.33	37.33
**Middle (2)**	Recording	57.39	42.44	0.74
	Model 2	4.6	19.71	4.27
**Ventricular (3)**	Recording	8.40	4.42	0.53
	Model 2	3.70	2.47	0.67

The value of each variable is given for both model and recorded ECG. P to R refers to the difference relative difference in magnitude between the P and R waves, and so on.

**Fig 8:**
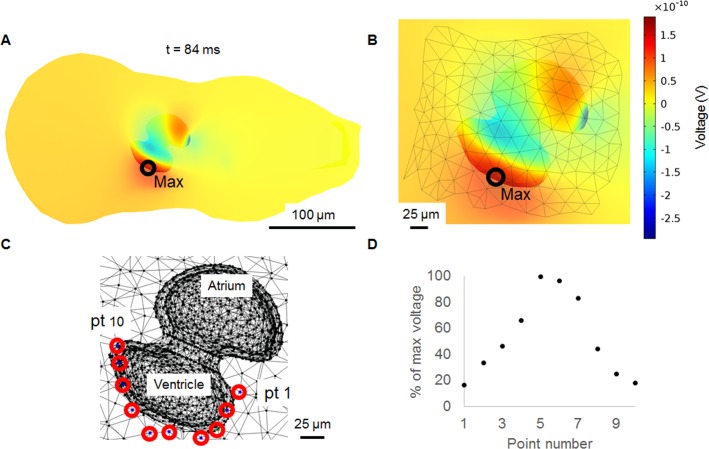
Maximum R wave voltage and R wave percentage change. The point at which the R wave is at its maximum at 0.84 s from the start of the simulation. A) zoomed out ventral view of the model with the point of maximum voltage intensity labelled, B) close-up ventral view with the maximum point labelled, C) close-up ventral view showing ten different points distributed around the ventricle as seen on the surface of the body, D) percentage of the maximum R wave voltage at each point.

As well as simulating the ECG, the model was useful in optimising recording positions. The maximum value of the R wave was determined by selecting the time at which R occurs (0.84 ms from the start of the simulation) in position 3 and then finding the maximum voltage on the surface of the body above the heart, in this case a point on the edge of the ventricle (**Fig 8**). Further investigation of the voltage distribution within the model revealed that the amplitude of the R wave decreased either side of point 5 (**Fig 8C and 8D**) around the edge of the ventricle. If a recording electrode was placed at the furthest points from the maximum (point 1 and point 10) less than 20% of the maximum R wave amplitude could be recorded across approximately 75 μm in either direction. Similarly, a decrease in amplitude was seen experimentally by moving the recording electrode across the surface of the zebrafish and measuring a change in amplitude of the signal [6].

## Discussion

The larval zebrafish ECG has previously been established as a viable tool for determining the effect of cardiac drug treatments [3]. Zebrafish larvae, however are relatively small which makes it difficult to achieve accurate and reproducible electrode positioning, which is important as electrode position affects the shape of the measured ECG. Therefore, in order to better understand how a measured ECG waveform is related to the structure of the heart its position within the larva and the position of the electrodes, a 3D model of a 3 days post fertilisation (dpf) larval zebrafish was developed to simulate cardiac electrical activity and investigate the voltage distribution throughout the body and simulate the resultant ECG. We have created the first electrical model of a zebrafish larval heart incorporating three-dimensional geometry of the body and heart, and simulated the ECG using bidomain equations. Our results show that the model can produce ECGs that show resemblance to real recordings, and is therefore of use in *in situ* investigations.

### Model production

The creation of the 3D geometry of the model body was aided by the ease in which the larval body can be imaged by simple bright field microscopy, with images captured from multiple angles by orientating the 3 dpf zebrafish relative to the objective lens. The heart geometry was more difficult to produce as it is located in a translucent sac, so fluorescent images were needed to produce a clear image that could be used to establish the geometry. As a working heart expands and contracts rhythmically, and the model only simulated electrical activity, a mid-point in the heartbeat cycle was chosen for replication and the chambers were approximated as being close to equal in volume.

### Continuum approximation

Cardiac propagation at the microscopic level is discontinuous due to the cellular nature of cardiac tissue [63]. Therefore, in order to produce the most realistic model of a heart each individual cell and the connections between them (gap junctions) would need to be modelled individually to form a discrete model. Gap junctions have a lower conductivity than cells, which reduces the overall conduction velocity of the activation wave [64]. Discrete models have previously been made for sections of cardiac tissue where the geometry is not as complex as an entire heart [65, 66]. The disadvantage of this approach is that it increases the computational load and the complexity of the model geometry. We used an alternative approach in this study and treated the heart as a single object approximating the discontinuous cellular structure as a continuum (as is used in the bidomain model) where the behaviour of individual cells averages out [27, 30]. This is the approach that is commonly taken when modelling the adult human heart as it contains a very large number of cells with complex geometry that is difficult to accurately reproduce; this approximation works well due to the large ratio between size of each cell and the whole heart. But for the developing zebrafish heart, which is much smaller, the cellular structure becomes more pronounced. A discrete model of the zebrafish larval heart is therefore desirable but was not feasible due to difficulty in determining cell positions/gap junction distributions and the computational load that would be required.

Generally, bidomain models are applied to the adult human heart or sections of tissue and the values used for both intracellular and extracellular conductivity are three sets of measurements [67–69] that show significant disagreement. The range of values measured is due to the difficulty in measuring these values, the difference in measurement methods and the variability in the composition of cardiac tissue [70, 71]. The activation waves produced using these parameters have conduction velocities that fall within the measured ranges [30] for bulk human tissue. However, if the size of the tissue is reduced to just a few cells or a single chain of cells the measured velocity decreases [64], due to the increased effect of gap junctions at the microscopic level. If an attempt is then made to model this system with the same conductivity parameters as in the larger case, the conduction velocity of the resulting waves will have the same value as for the larger case and will not agree with measurements. This is a limitation of the continuum approximation as the measured conductivities are mean values, with the intracellular conductivity encompassing the conductance of the gap junctions [72], but they do not account for the structural change that occurs with small amounts of cells. Therefore, to effectively model this situation either gap junctions need to be introduced or the conductivity parameters need to be changed, as was done in the model presented here.

In addition to the continuum approximation limitations the reason for reducing the conductivity, compared to a human heart model, is due to the large difference in conduction velocity between the human and zebrafish hearts. Modelling a small section of human heart tissue would not require such a large reduction as the intrinsic velocity is higher to start with. Conduction in developing hearts is slow as the faster conducting pathways and tissue require maturation. The larval zebrafish heart does not develop a conduction system until after 3 dpf [9] so propagation is initially slow, e.g. the adult zebrafish heart conduction velocity is over 100 mm/s [73] whereas in the larval heart it is around 10 mm/s [61]. There are no available measurements of the conductivity of zebrafish larval hearts but if there were such measurements available they would likely be much lower than that of the adult human heart. This required introduction of a conductivity scaling factor to reduce conduction velocity to match recordings, on the assumption that provided the overall morphology of the heart and body is realistic and activation of the heart can be approximated as an excitation wave, then scaling conductivity to alter the conduction velocity of the wave is a valid approach. We were not attempting to model the finer details of cardiac conduction but the larger scale ECG, which can be determined from depolarisation and repolarisation wave progression.

As stated previously the surface-volume-ratio and membrane capacitance also have an effect on the conduction velocity. The size of the zebrafish heart and cells mean that the surface-to-volume ratio is much higher than for human cells which leads to a lower conduction velocity. An estimate of surface-to-volume ratio was made based measured values of area and volume [8] but any change in this value would result in different value of conductivity to achieve the same conduction velocities. The same is true for capacitance, the small cell size likely results in a smaller capacitance than the value used in this work.

The relationship between conductivity and conduction velocity can be illustrated by a separate 2D model (**S3 Fig**) consisting of three blocks, one for the sinoatrial region, one for the atrium and one for the body. Using this geometry the wave velocity can be determined from a known distance. Activation was initiated at the sinoatrial block then the conduction velocity of the propagated wave was determined between two points in the centre of the block. A range of conductivity values were then used with the wave speed calculated for each, as can be seen in **S3 Fig**.

Real heart tissue is highly anisotropic with the large majority of activation travelling along the length of cardiac chains of cells or fibres [30], and also in the zebrafish larval heart where the velocity was direction-dependent [61]. As this model is a first approximation, future refinements such as including anisotropic geometry may account for the discrepancies between the current model and the experimental data. Despite this the key features of the measured ECGs have been reproduced by the current model.

### Action potential comparison

The APD and magnitude are in good agreement with the measured values as they were the simplest properties to change in the model, being controlled mainly by a single parameter for each (e for the duration and A for the magnitude). The upstroke and downstroke values showed less agreement, especially for the downstroke of the atrium and upstroke of the AV band, where the model APs were found to be half as steep as the recorded values. The gradients of the upstroke and downstroke could not be finely controlled by the model parameters, but for the ventricle was within 20%. Combined with the APD and magnitude similarities, this shows that the model ventricle AP had the best fit, followed by the atrioventricular band AP.

### ECG comparison

One of the main differences between the modelled (based on the measured APs) and recorded ECGs is the difference in QT interval, which should be approximately equal to the ventricle APD. This was assumed to be due to the different conditions that the AP and ECG measurements were performed under (temperature and zebrafish age). If a ventricle AP was recorded at 28°C for a 3 dpf zebrafish, the APD should be shorter than at room temperature and 2 dpf. This could account for the difference in APD between the measured ventricle APD and the QT interval in the measured ECG. Therefore, the model was changed to reflect this and a similar QT interval to the measured ECGs was achieved. In the future the model APs could be fitted to measurements of APs that were recorded under the same conditions as the ECGs. The PR interval was found to be shorter in the model for position 2 and position 3. Shorter PR intervals indicate that the delay caused by the atrioventricular band was not long enough, which could be accommodated by decreasing conduction velocity or extending the width of the atrioventricular band region. The increase in PR interval for position 1 was most likely due to the position of the Q wave being distorted by the increased atrial activity that was seen in the model ECGs; this created a peak which overwhelmed the QRS complex moving the position of the Q wave to an apparent later time and increasing the PR interval.

The results from the model and the measured ECGs were consistent with how the activation wave has been shown to travel through the zebrafish heart [61]. There is some debate in the literature regarding the presence of a ventricular conduction system. Such a system could result in apex-to-base [74] (edge of ventricle towards AV band) rather than base-to-apex [75] activation. The activation map [61] shows only base-to-apex activation and based on the model results, apex-to-base activation would invert the polarities of the R and T waves which wouldn’t agree with the measured ECGs. Therefore, there is no evidence to suggest that a ventricular conduction system is present at 3 dpf but one could still develop at a later stage.

The model larval zebrafish ECGs showed appropriate polarity based on the progression of the depolarisation wave as it moved away from positions 1 & 2 and towards position 3, and repolarisation moved away from position 3 and towards position 1. The model ECG from the atrial position (position 1) was found to be different to the recording, which showed a large amount of ventricular activity with little activity from the atrium; the model showed equal amounts of atrial and ventricular repolarisation. This is most likely due to the difference in muscle mass between the thicker ventricular and thinner atrial walls within the zebrafish, which was not replicated within the model. The atrial signal would likely be dominated by the signal strength from the ventricle, as in humans where atrial repolarisation is not normally seen in the ECG as ventricular depolarisation (R wave) occurs at the same time. The model ECG peaks showed good agreement with the polarity of the measured ECG peaks, with each peak visible and with the same polarity, especially for position 2 and 3. The width of the peaks showed less agreement, with the model R waves being significantly narrower than the recording. The P wave duration was found to be closer to the measured value but still shorter, possibly due to the gradient of the model ventricle AP upstroke which affected the width of the ECG peaks in the model. The wide ECG peaks seen in the recordings could be due to decreased AP gradient at 3 dpf. In the model a steeper upstroke was found to result in a narrower ECG peak and a shallower upstroke resulted in a wider peak, although the model did not allow for fine control of the upstroke gradient. Another factor in the peak width difference is that the ECGs from the model are from a point on the surface of the body which is effectively a perfect electrode with no area averaging effects (spatial filtering), noise, interface or electrode properties that could contribute to the disparity in peak widths.

The model can predict the result APD prolongation has on the polarity of the T wave. The reduction of the APD for model 2 effectively means that the model 1 APD is prolonged. As can be seen from the ECG comparisons, both versions of the model give the same T wave polarity. This was to be expected as there is only one ventricle region within the model and repolarisation occurs in the same order as depolarisation (but with reversed polarity) for both models. Prolonging the APD therefore only has the effect of delaying the T wave in the model. This is not the case in the human ventricle as the inner muscle (endocardium) has a longer APD than the outer muscle (epicardium) [30] so changes in APD can affect the direction of the repolarisation and hence the T wave polarity. There is evidence that different tissues are present in the larval zebrafish ventricle [8] but there are no measurements of any differences in APD between the regions to determine if they differ similarly as they do in the human heart. The polarity of the measured ECGs suggest that it is not the case (at least at 3 dpf), as the T wave polarity is consistent with inner to outer repolarisation as outer to inner repolarisation would result in the reverse T wave polarity.

The relative peak amplitude disagreements between the model and measured values could be due to the distorted position of the Q wave, AP shape and tissue volume or electrode position differences. The R wave amplitude decreased either side of a maximum point. The drop was not so severe that a 2 μm diameter electrode could not be positioned to achieve a good signal, but larger electrodes would require finer positioning due to the greater effect of spatial filtering. Further experiments would be needed to determine how accurate the model prediction is.

## Conclusions

### Summary

A 3D electrical model of the zebrafish larvae body and heart was constructed that simulates the ECG of the larval zebrafish using the bidomain model. Electrical activation is initiated in the sinoatrial region where an AP propagates through the heart as a wave to the ventricle at the same rate as measured velocities. The model APs were based on those measured from a 2 dpf zebrafish. From the resulting voltage distribution, the ECG was investigated at three different positions and compared to measurements. The key features (P wave, R wave, T wave) of the ECG were reproduced by the model along with the polarity of each peak

### Limitations and future work

The geometry of the heart affects the shape of the ECG. Whilst the geometry of the body and heart was made as realistic as possible, it is not completely accurate to the 3 dpf zebrafish larva. Finer imaging techniques could be used to improve the geometry further [46]. Also, a continuum model is not ideal for the larval zebrafish heart due to its small size, therefore a discrete model could be introduced. The Fitzhugh-Nagumo equations were unable to replicate the exact shape of the zebrafish APs so a more realistic model could be introduced similar to [22] with parameters measured from the zebrafish, allowing drug and mutant effects on the ECG to be investigated. The real heart is not a fixed electrical source, and contractions have been shown to affect the shape of the ECG [20]. Movement of the zebrafish heart could be incorporated using images to determine displacement or a full mechanical model could be used based on further measurements. These different improvements in the model are aimed at generating simulated ECGs which are as close as possible those measured with electrodes placed on the skin of a real zebrafish larva. In implementing them we would also be able to improve our understanding of how the structure of the cardiac system determines the detailed waveform of the ECG. Further development would enable us to incorporate features associated with the diseased heart and hence assist in the interpretation of larval zebrafish ECGs in these conditions.

## Supporting Information

S1 Fig Effect of changing parameter ‘e’ on APD. A range of values of e were used then the resulting APD was measured. The result is plotted on the graph.
(TIF)

S2 Fig Model 1 and 2 APs. Model 2 APs are a result of reducing the APD so that the model ECG QT interval is reduced to the measured QT interval.
(TIF)

S3 Fig Conduction velocity and conductivity relation. (A) 2d block model. 3 regions consisting of body, sinoatrial and atrium. Points 1 and 2 were used to determine the conduction velocity. (B) The relationship between sigma (the conductivity or diffusion coefficient) and the resulting conduction velocity of the wave. The first horizontal line shows the slowest velocity that occurs in the human heart in AV region and the bottom limits show the measured velocities in a 3 dpf zebrafish ventricle [61].
(TIF)

S1 File Bidomain equation derivation. (DOCX)

S2 File Progression of Vm over time. Animated GIF of progression of Vm as a surface plot over time (0 to 0.5 s). Maximum (top right) and minimum voltages (bottom right) are shown. Colour scale is voltage in volts.
(GIF)

S1 Table Combined parameter table of all parameters. (DOCX)
