# Supplementary figures and images for: 3D Finite Element Electrical Model of Larval Zebrafish ECG Signals
Source: PLoS One. 2016 Nov 8;11(11):e0165655. doi: 10.1371/journal.pone.0165655 (PMC5100939; doi:10.1371/journal.pone.0165655)

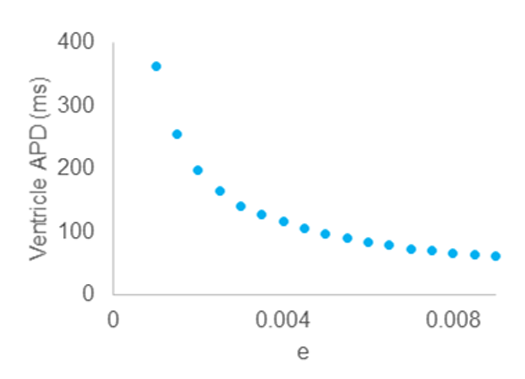

Supplement: S1 Fig — A range of values of e were used then the resulting APD was measured. The result is plotted on the graph. (TIF) [file pone.0165655.s001.tif]

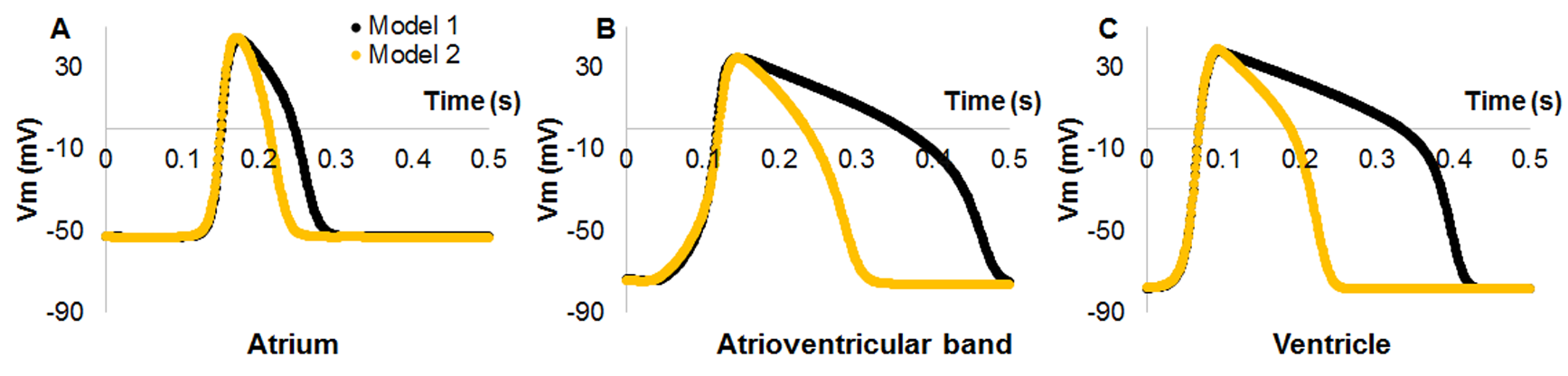

Supplement: S2 Fig — Model 2 APs are a result of reducing the APD so that the model ECG QT interval is reduced to the measured QT interval. (TIF) [file pone.0165655.s002.tif]

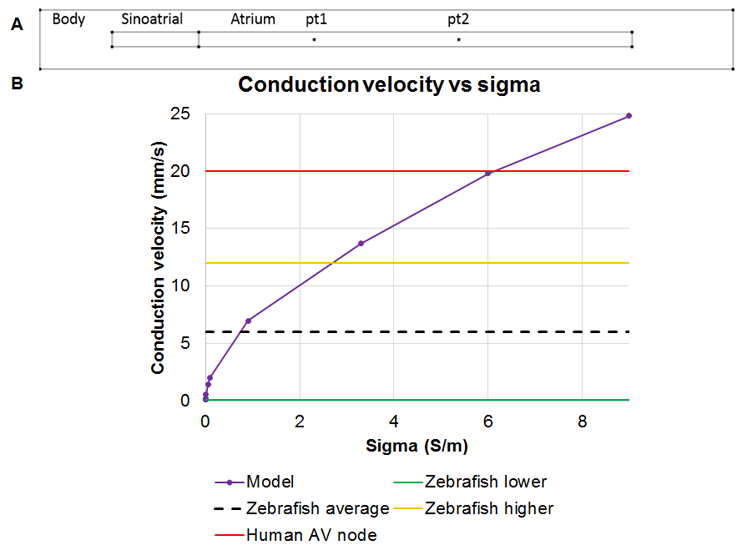

Supplement: S3 Fig — (A) 2d block model. 3 regions consisting of body, sinoatrial and atrium. Points 1 and 2 were used to determine the conduction velocity. (B) The relationship between sigma (the conductivity or diffusion coefficient) and the resulting conduction velocity of the wave. The first horizontal line shows the slowest velocity that occurs in the human heart in AV region and the bottom limits show the measured velocities in a 3 dpf zebrafish ventricle [61]. (TIF) [file pone.0165655.s003.tif]

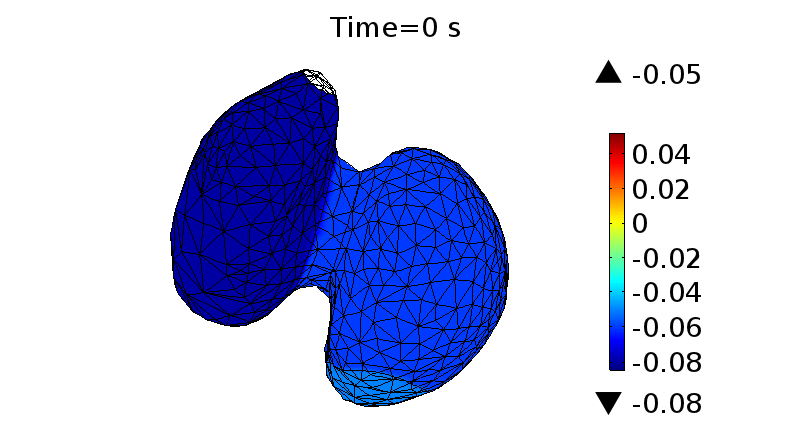

Supplement: S2 File — Animated GIF of progression of Vm as a surface plot over time (0 to 0.5 s). Maximum (top right) and minimum voltages (bottom right) are shown. Colour scale is voltage in volts. (GIF) [file pone.0165655.s005.gif]
