# Supplementary material for: 3D Finite Element Electrical Model of Larval Zebrafish ECG Signals
Source: PLoS One. 2016 Nov 8;11(11):e0165655. doi: 10.1371/journal.pone.0165655 (PMC5100939; doi:10.1371/journal.pone.0165655)
Supplement: S1 File — (DOCX) [file pone.0165655.s004.docx]

**Supplementary Text 1**

The bidomain equations are derived as follows [23] starting with the definition of the potential difference across the cell membrane boundary (transmembrane potential):

|  | $V_{m}=V_{i}- V_{e}$ | (1) |
| --- | --- | --- |

Current is assumed to only flow between the extracellular region and the body according to the defined boundary conditions. Ohms law states:

|  | $\boldsymbol{J}= \sigma\boldsymbol{E}$ | (2) |
| --- | --- | --- |

Where J is the current density, σ is conductivity and E is the electric field. Using the quasistatic approximation:

|  | $\boldsymbol{E}= -\nabla V$ | (3) |
| --- | --- | --- |

Where V is the voltage. Then for the two domains combining (2) and (3):

|  | $\boldsymbol{J}_{i}= -\sigma_{i}\nabla\cdot V_{i}$ | (4) |
| --- | --- | --- |
|  | $\boldsymbol{J}_{e}= -\sigma_{e}\nabla\cdot V_{e}$ | (5) |

Any current that leaves one domain must flow into the other due to current conservation therefore the change in current density between the two domains is equal in magnitude and opposite in sign:

|  | ${-\nabla\boldsymbol{J}}_{i}= \nabla\boldsymbol{J}_{e}= {A_{m}I}_{m}$ | (6) |
| --- | --- | --- |

Where I_m_ is the transmembrane current, given by:

|  | $I_{m}= C_{m}\frac{\partial V_{m}}{\partial t}+ i_{ion}$ | (7) |
| --- | --- | --- |

Where C_m_ is capacitance per unit area and i_ion_ is the ionic current. Then, combining (13) (14) and (6) gives:

|  | $\nabla\cdot\left( -\sigma_{i}\nabla V_{i} \right)= {A_{m}I}_{m}$ | (8) |
| --- | --- | --- |
|  | $\nabla\cdot\left( -\sigma_{e}\nabla V_{e} \right)= {-A_{m}I}_{m}$ | (9) |
