# Supplementary material for: 3D Finite Element Electrical Model of Larval Zebrafish ECG Signals
Source: PLoS One. 2016 Nov 8;11(11):e0165655. doi: 10.1371/journal.pone.0165655 (PMC5100939; doi:10.1371/journal.pone.0165655)
Supplement: S1 Table — (DOCX) [file pone.0165655.s006.docx]

| **Parameter** | **SAR** | **Atrium** | **AV Band** | **Ventricle** | **Body** | **Chambers** |
| --- | --- | --- | --- | --- | --- | --- |
| **a** | -0.5 | 0.13 | 0.13 | 0.13 | - | - |
| **b** | 0.4 | 0 | 0 | 0 | - | - |
| **c1** | 0.182 | 0.572 | 0.234 | 0.572 | - | - |
| **c2** | 1 | 0.5 | 0.7 | 0.5 | - | - |
| **d** | 1 | 1 | 1 | 1 | - | - |
| **e** | 0.0001 | 0.004 | 0.00032 | 0.0011 | - | - |
| **A** | 0.2 | 0.1088 | 0.1085 | 0.1205 | - | - |
| **B** | -0.135 | -0.053 | -0.07 | -0.075 | - | - |
| **k** | 1000 | 1000 | 1000 | 1000 | - | - |
| **Am (m^-1^)** | 90000 | 90000 | 90000 | 90000 | - | - |
| **Cm (Fm^-2^)** | 0.01 | 0.01 | 0.01 | 0.01 | - | - |
| **Vi** (V) | -0.05 | -0.053 | -0.0585 | -0.075 | - | - |
| **Ve** (V) | 0 | 0 | 0 | 0 | - | - |
| **u** | 0 | 0 | 0 | 0 | - | - |
| **σe** (Sm^-1^) | 1.81E-05 | 1.81E-05 | 2.43E-06 | 1.55E-05 | - | - |
| **σi** (Sm^-1^) | 1.81E-05 | 1.81E-05 | 2.43E-06 | 1.55E-05 | - | - |
| **σb** (Sm^-1^) | - | - | - | - | 0.2 | - |
| **σ**c (Sm^-1^) | - | - | - | - | - | 0.7 |

Key: SAR =sinoatrial region; Atrium = atrial wall muscle; AV band = atrioventricular band; Ventricle = ventricle wall muscle; Body = zebrafish model body; Chambers = heart chambers
